# The Afghan War Scandals

**Published:** 1919-08-23

**Authors:** 


### The Hospital

The Workers' Newspaper of Administrative Medicine and Institutional Life, Administration, National Insurance and Health.

No. 1733, Vol. LXVI. SATURDAY, AUGUST 23, 1919. PRICE TWOPENCE

### THE AFGHAN WAR SCANDALS.

Last week we dealt with cholera and sickness on the Afghan Frontier. There has been much mismanagement and incompetence displayed by the Indian Government authorities, and this has been aggravated by the issue of the White Paper containing some of the telegrams which have passed between the India Office and the Indian Government. No one can doubt this exhibition of considerable incompetence, and the official plea which aims at placing the blame on a state of war, and the depletion in consequence of medical supplies, in the known circumstances of to-day, cannot be accepted with patience. If we may judge by the delay in the answers sent to the telegrams of the Secretary of State for India, there must still be an absence of grip and vigour attaching to the authorities in India, which calls for prompt action and immediate remedy. Mr. Montagu has shown commendable interest in the reform and betterment of the condition and circumstances of the medical organisation in India. We hope, however, that the omission of the questions relating to the position and pay of British officers in the Indian Army and Medical Service will be immediately put right.

The correspondence regarding the medical arrangements and comforts for the troops on the North-West Frontier was issued late on Saturday, the 16th instant. A perusal of this White Paper illustrates the dilatory character of the way in which serious matters like those included in this correspondence are treated by the Government of India, the answers of which to the Secretary of State's telegrams are not all included, for the sufficient reason that they have not yet been received. An important telegram from London requesting evidence of the truth of statements appearing in the Indian newspapers was not fully replied to by the Viceroy, Army Department, until August 5. The *Times* properly asks: "Is it a fact that the War Office depleted India of medical stores for Mesopotamia? And, if so, why were they not replaced?" These pertinent questions go to the root of the whole matter. The state of dilatory ineptitude and want of grip is lamentable in the circumstances. This White Paper, in fact, cannot be taken as a defence of the mismanagement which it demonstrates has occurred.

It had been anticipated and expected that there would be serious trouble on the Afghan Frontier, but the chief explanation put forward is that it was impossible to supervise arrangements for an immediate supply of pure water throughout the terrain, involving six separate lines of operations, and covering a front approximately 500 miles long; no complete or adequate protective measures were initiated before an outbreak of cholera occurred; and when the attempt was made to provide supplementary piped water the work was hampered from the commencement by shortage of pumps and pipes demanded from England, Mesopotamia, and elsewhere. Could self-condemnation of authorities by themselves go further than this?

It would appear further that, although Overseas Base Reserves were despatched to the frontier within forty-eight hours of mobilisation, on being ordered "in accordance with our pre-arranged plans" the authorities had to proceed to "the immediate purchase of articles necessary to complete rations to the present scale." Again, when the Afghan situation developed into a crisis and was at once followed by the defection of our Frontier Militia together with the threat of a rebellion in Peshawar itself, "not only had the advance echelons of the Field Army to be diverted to replace the Militia, but they had actually to preserve order in the districts." Then follows the amazing confession that, the Indian authorities, "instead of being able to proceed on a deliberate system of rapid concentration," found themselves "forced at once to send troops to every part of the frontier, and so reverse their pre-arranged plan of concentration, which provided that the despatch of stores and equipment should precede that of troops." Small wonder that the Secretary of State for India should feel called upon to telegraph under date of August 13 as follows: "Specific allegations have been made that for a few days there were no arrangements made for feeding officers, no sheets or basins, that an officer's private basin was seized and all patients washed in it, and that the service of food was bad. I cannot reconcile this with Kohat's statement that there was no deficiency in hospital furniture and sheets. Please make further inquiry and report for inclusion in Blue-book this week." The reply to this was not received from India in time for inclusion in the White Paper, nor was a reply to the Secretary of State's telegram of August 7 regarding the "necessity for pipe line for water to camp site at Ali Masjid, but nothing done until outbreak of cholera recently."

The White Paper brings out one gratifying circumstance which bears testimony to the high efficiency and untiring work of the Director of Medical Services, Khyber Front, and his staff. Between May 15 and May 17, which included the dates between which the most severe fighting took place, approximately, 322 of all ranks of British and Indian were wounded on the Khyber line. The majority of the British wounded were in Station Hospital, Peshawar, within a few hours of the action, and some actually transferred by ambulance train to Rawal Pindi within the course of the day. The splendid efficiency of the R.A.M.C., owing to the resourceful way in which the members of this Corps in command, and their officers under them, have discharged their duties under most trying circumstances on many battlefields during the war, is here demonstrated to be the one bright spot in the arrangements on the North-West Frontier of India, where several things have happened in other directions which must entail full inquiry, complete investigation, and detailed reports. We defer further comment and suggestions until the additional White Paper is issued containing the Viceroy's, Army Department, answer to the two telegrams of the Secretary of State for India already referred to.

